# Associations and Interactions between Heavy Metals with White Blood Cell and Eosinophil Count

**DOI:** 10.7150/ijms.68945

**Published:** 2022-01-09

**Authors:** Chao-Hsin Huang, Chieh-Yu Hsieh, Chih-Wen Wang, Hung-Pin Tu, Szu-Chia Chen, Chih-Hsing Hung, Chao-Hung Kuo

**Affiliations:** 1 Department of post baccalaureate medicine, Kaohsiung Medical University, Kaohsiung, Taiwan; 2 Department of Internal Medicine, Kaohsiung Municipal Siaogang Hospital, Kaohsiung Medical University, Kaohsiung, Taiwan; 3 Division of Hematology and Oncology, Department of Internal Medicine, Kaohsiung Medical University Hospital, Kaohsiung Medical University, Kaohsiung, Taiwan; 4 Division of Hepatobiliary, Department of Internal Medicine, Kaohsiung Medical University Hospital, Kaohsiung Medical University, Kaohsiung, Taiwan; 5 Department of Public Health and Environmental Medicine, School of Medicine, College of Medicine, Kaohsiung Medical University, Taiwan; 6 Division of Nephrology, Department of Internal Medicine, Kaohsiung Medical University Hospital, Kaohsiung Medical University, Kaohsiung, Taiwan; 7 Faculty of Medicine, College of Medicine, Kaohsiung Medical University, Kaohsiung, Taiwan; 8 Research Center for Environmental Medicine, Kaohsiung Medical University, Kaohsiung, Taiwan; 9 Department of Pediatrics, Kaohsiung Medical University Hospital, Kaohsiung Medical University, Kaohsiung, Taiwan; 10 Department of Pediatrics, Kaohsiung Municipal Siaogang Hospital, Kaohsiung Medical University, Kaohsiung, Taiwan; 11 Division of Gastroenterology, Department of Internal Medicine, Kaohsiung Medical University Hospital, Kaohsiung Medical University, Kaohsiung, Taiwan

**Keywords:** heavy metals, white blood cell, eosinophil count, interaction

## Abstract

The accumulation of heavy metals in the body has been associated with an elevated immune response. The aim of this study was to investigate the associations among heavy metals and white blood cell (WBC) and eosinophil count in the general population in southern Taiwan. We also explored the interactions and synergetic effects of heavy metals on WBC and eosinophil count. We conducted a health survey in the general population living in southern Taiwan between June 2016 and September 2018. Seven heavy metals were measured: blood lead (Pb), and urine cadmium (Cd), copper (Cu), nickel, arsenic (As), chromium and manganese (Mn). A total of 2,447 participants were enrolled. In multivariable analysis, high concentrations of Pb (log per 1 mg/L; coefficient β, 0.332; *p* = 0.005) and Cu (log per 1 μg/dL; coefficient β, 0.476; *p* < 0.001) were significantly associated with a high WBC count. In addition, high concentrations of Pb (log per 1 mg/L; coefficient β, 0.732; *p* < 0.001), As (log per 1 μg/L; coefficient β, 0.133; *p* = 0.015), Cu (log per 1 μg/dL; coefficient β, 0.181; *p* = 0.018), and Cd (log per 1 μg/L; coefficient β, 0.139; *p* = 0.002) were significantly associated with a high eosinophil count. Further, the effect of interactions between Pb and As (coefficient β, 0.721; *p* = 0.029) and Mn and Cu (coefficient β, 0.482; *p* = 0.018) on WBC count, and As and Cu (unstandardized coefficient β, 0.558; *p* = 0.002) on eosinophil count were statistically significant. In conclusion, the heavy metals Pb, As, Cu, and Cd were associated with WBC and eosinophil count. In addition, synergistic effects of heavy metal poisoning on the association with WBC and eosinophil count were also observed.

## Introduction

Environmental contamination of heavy metals has detrimental effects on human health and has attracted increasing attention worldwide. Taiwanese individuals especially were suggested to have higher level of heavy metal in blood such as Pb, Hg, and Cd were found in Taiwanese individuals compared to those of the Americans 1, indicating a heavy metal-related health burden in Taiwan.

Lead (Pb), manganese (Mn), copper (Cu), arsenic (As), and cadmium (Cd) are among the most common heavy metal pollutants found in industrial countries. For example, the emergence of the endemic “Blackfoot disease” in southwestern Taiwan in the early 20th century which caused peripheral microvascular disease manifesting as progressive gangrene of the feet, was found to be caused by high environmental concentrations of As 2. In addition, another study found higher levels of mercury and Cd in Taiwanese subjects than in other populations 3, and Cd has been associated with renal damage, cardiovascular disease, impaired neural function, and osteoporosis 4.

Exposure to heavy metals can be through the intake of water, rice and fish 5. Lifelong exposure to heavy metals can result in multiple organ failure, which is related to dysregulation of immune function and genomic instability. Interestingly, different levels of heavy metals can exert opposite effects on immune function 6. The immunomodulatory effect of heavy metal poisoning has been explored in animal studies 7-9, however associations between some heavy metals and immune responses in humans are still unclear. Therefore, the aim of this study was to investigate the relationships among heavy metals including blood Pb and urine Cd, Cu, nickel (Ni), As, chromium (Cr) and Mn with white blood cell (WBC) and eosinophil count in the general population in southern Taiwan. The effects of interactions among these heavy metals on WBC and eosinophil count were also explored.

## Study Subjects

Subjects living in southern Taiwan who participated in a health survey from June 2016 to September 2018 were enrolled in this study. The survey was promoted through advertisements. A total of 2447 participants (977 males and 1470 females) were included in this study, with a mean age of 55.1±13.2 years. All participants signed informed consent forms before being enrolled into this study, which was approved by the Institutional Review Board of Kaohsiung Medical University Hospital (number: KMUHIRB-G(II)-20190011).

## Materials and methods

### Medical, Demographic and Laboratory Variables

During the health survey, the sex, age, height and weight of each participant were recorded, along with systolic blood pressure (SBP) and diastolic blood pressure (DPB). In addition, each participant underwent a physical examination performed by an experienced physician, during which clinical histories of hypertension and diabetes mellitus (DM) were recorded. The following baseline data were also recorded: fasting glucose, triglycerides, total, high-density lipoprotein (HDL)- and low-density lipoprotein (LDL)-cholesterol, WBC count, hemoglobin, platelets, eosinophil count, estimated glomerular filtration rate (eGFR), uric acid, and body mass index (BMI; kg/m^2^). The eGFR was calculated using the Chronic Kidney Disease Epidemiology Collaboration (CKD-EPI eGFR) equation 10.

### Measurements of Heavy Metals in Urine and Blood

We measured the concentrations of blood Pb and urine Cd, Cu, Ni, As, Cr and Mn using graphite furnace atomic absorption spectrometry (ICP-MS, NexION 300 Series, Perkin Elmer). The National Institute of Environmental Research has published details of the instrumental analysis.

### Statistical Analysis

The statistical analysis was performed with SPSS version 19.0 for Windows (SPSS Inc. Chicago, USA). Percentages, means±standard deviations, or medians (25^th^-75^th^ percentiles) were used to describe eosinophil count, triglycerides and the seven heavy metals. The participants were classified into three groups according to the tertile of WBC count as follows: tertile 1, < 5.24*10^3^/μL; tertile 2, 5.24-6.49*10^3^/μL; and tertile 3, ≥ 6.49*10^3^/μL. One-way analysis of variance (ANOVA) followed by Bonferroni-adjusted post hoc test were used for multiple comparisons among groups. Multivariable linear regression analysis was used to identify associations between the heavy metals and WBC and eosinophil count. The natural logarithm was used for all heavy metal measurements. A generalized linear model was used to examine the effects of interactions among the studied heavy metals on WBC and eosinophil count. The LOESS procedure (a nonparametric technique used to estimate regression surfaces) was applied to illustrate the synergistic effects of heavy metals on WBC and eosinophil count using SAS software (version 9.4, SAS Institute, Cary, NC, USA). A *p* value of less than 0.05 was considered to indicate a statistically significant difference.

## Results

Comparisons of the clinical characteristics of the participants according to tertile of WBC count are shown in Table 1. Compared to the participants in tertile 1, those in tertile 3 were younger, predominantly male, had a higher prevalence of DM, and higher BMI, SBP, DBP, fasting glucose, triglycerides, LDL-cholesterol, WBC count, hemoglobin, platelets and uric acid, and lower HDL-cholesterol. In addition, the participants in tertile 3 had higher levels of urine As, Cu and Cd.

### Associations among the Heavy Metals and WBC Count

We next investigated associations among the seven heavy metals and WBC count using multivariable linear regression analysis (Table 2). After adjusting for each heavy metal, sex, age, BMI, hypertension, DM, fasting glucose, log triglycerides, total cholesterol, LDL-cholesterol, HDL-cholesterol, SBP, DBP, eGFR, uric acid, hemoglobin, and platelets, the participants with high concentrations of blood Pb (log per 1 mg/L; unstandardized coefficient β, 0.332; 95% confidence interval [CI], 0.101 to 0.562; *p* = 0.005) and urine Cu (log per 1 μg/dL; unstandardized coefficient β, 0.476; 95% CI, 0.232 to 0.721; *p* < 0.001) were significantly associated with a high WBC count.

### Associations among the Heavy Metals and Eosinophil Count

We then investigated associations among the seven heavy metals and eosinophil count using multivariable linear regression analysis (Table 3). After adjusting for each heavy metal, sex, age, BMI, hypertension, DM, fasting glucose, log triglycerides, total cholesterol, LDL-cholesterol, HDL-cholesterol, SBP, DBP, eGFR, uric acid, hemoglobin, and platelets, the participants with high concentrations of blood Pb (log per 1 mg/L; unstandardized coefficient β, 0.732; 95% CI, 0.594 to 0.869; *p* < 0.001), urine As (log per 1 μg/L; unstandardized coefficient β, 0.133; 95% CI, 0.025 to 0.240; *p* = 0.015), urine Cu (log per 1 μg/dL; unstandardized coefficient β, 0.181; 95% CI, 0.031 to 0.330; *p* = 0.018), and urine Cd (log per 1 μg/L; unstandardized coefficient β, 0.139; 95% CI, 0.052 to 0.226; *p* = 0.002) were significantly associated with a high eosinophil count.

### Effect of Interactions among the Heavy Metals on WBC and Eosinophil Count

**Figure 1:**
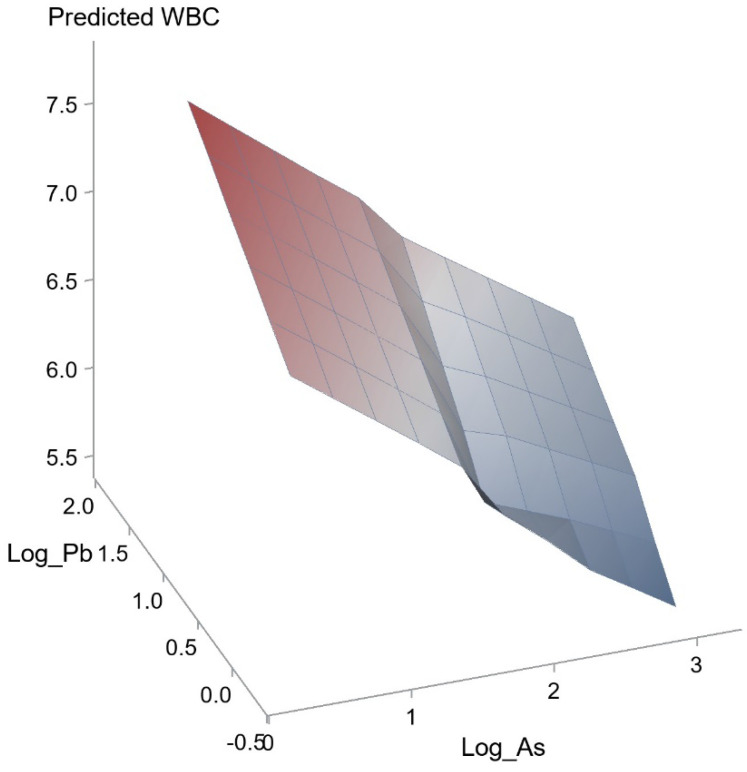
Synergistic effect of Pb and As on WBC. The interaction between Pb and As on WBC was statistically significant (*p* = 0.029).

**Figure 2:**
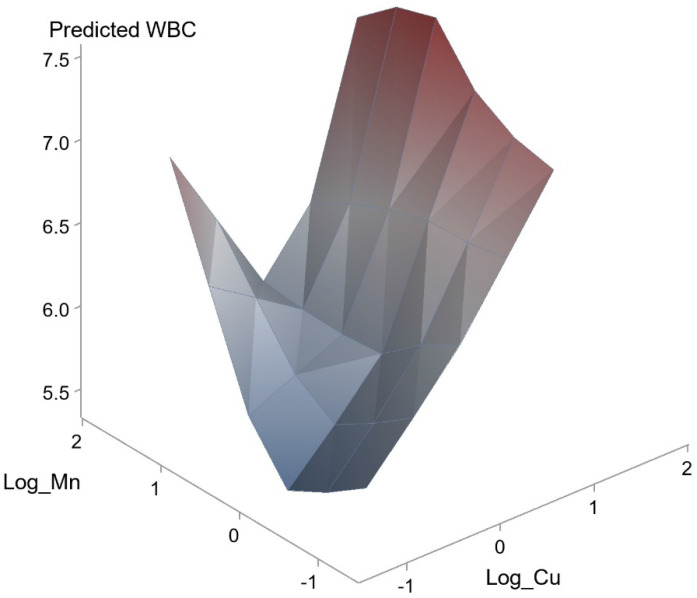
Synergistic effect of Mn and Cu on WBC. The interaction between Mn and Cu on WBC was statistically significant (*p* = 0.018).

We further performed analysis of the effects of interactions among the seven heavy metals on WBC and eosinophil count was conducted using a generalized linear model. The effects of interactions between Pb and As (unstandardized coefficient β, 0.721; *p* = 0.029) and Mn and Cu on WBC count (unstandardized coefficient β, 0.482; *p* = 0.018) were statistically significant. However, the effects of interactions of other combinations did not achieve statistical significance. Figure 1 and 2 illustrate the synergistic effects of Pb and As and Mn and Cu on WBC count. Synergistic effects of Pb and As and Mn and Cu on the association with WBC were observed. This was a combined analysis trying to model the effect of heavy metals on WBC count based on levels of Pb and As (Figure 1; *p* for interaction = 0.029) or levels of Mn and Cu (Figure 2; *p* for interaction = 0.018).

**Figure 3:**
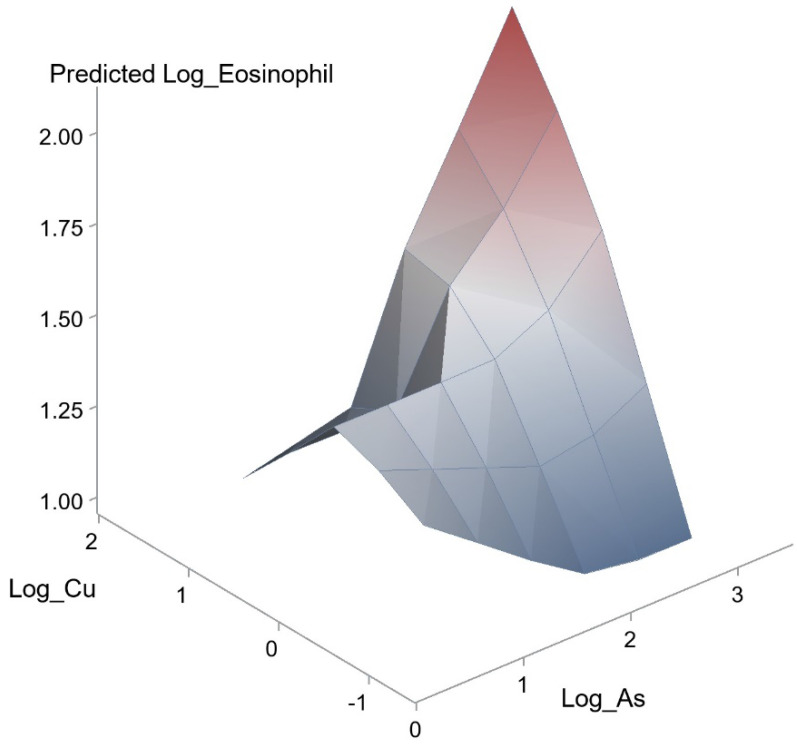
Synergistic effect of As and Cu on eosinophil count. The interaction between As and Cu on eosinophil count was statistically significant (*p* = 0.002).

In addition, the effects of interactions between As and Cu (unstandardized coefficient β, 0.558; *p* = 0.002) on eosinophil count was statistically significant. A synergistic effect of As and Cu on the association with eosinophil count was also observed (Figure 3).

## Discussion

In this study, we investigated associations between immune function and heavy metals among 2,447 Taiwanese participants. Overall, the results showed that high concentrations of Pb and Cu were associated with a high WBC count, and those high concentrations of Pb, As, Cu and Cd were associated with a high eosinophil count. In addition, the results showed positive interaction effects between Pb and As and Mn and Cu on WBC count, and As and Cu on eosinophil count.

There are several important findings in this study. First, a high concentration of blood Pb was correlated with high WBC and eosinophil count. Further, a synergistic effect of Pb and As on WBC count was observed. Pb poisoning is well-known to cause both peripheral and central neurological disorders such as wrist drop, Pb encephalopathy, and impaired coordination 11, 12, which in turn have been associated with immunomodulation 13, 14. The severity of symptoms has been reported to be dose- and time-dependent, and a level higher than 10 μg/dl in the blood is cause for concern 15. The role of Pb in immunomodulation has been extensively studied. Struzynska et al. 7 investigated immune responses to Pb poisoning in immature rat brains by continuously injecting 15 mg/kg lead acetate into 15-day-old pups for two weeks. The results showed elevated levels of interleukin (IL)-1β, tumor necrosis factor (TNF)-α, and IL-6 in different areas of the brain, implying increased immunoreactivity under Pb exposure 7. In another study, Farkhondeh et al. 8 investigated immune reactions to different concentrations of Pb exposure in guinea pigs. In their study, the guinea pigs inhaled 0.1 M, 0.2 M and 0.4 M of aerosol Pb for 1 hour twice a week for two weeks, after which blood samples were collected and analyzed. The results showed elevated levels of total protein, total WBCs, histamine, and eosinophils after Pb exposure, supporting the proinflammatory effect of Pb poisoning 8. In addition, Hemdan et al. 16 conducted an in vitro study to investigate interactions between Pb exposure and cytokine secretion. The results showed elevated levels of IL-4, IL-6 and IL-10, indicating activation of a T helper 2 cell response and the potential activation of eosinophil production 16. The results of the present study are consistent with the previous findings that Pb exposure is related to increased WBC and eosinophil count. In addition, our results also suggested that As did not have a significant effect on WBC, but that As and Pb had a significantly positive interaction effect on WBC. This finding suggests that Pb may have a synergistic effect with As on immune activity, which may be caused by the dual effects of Pb and As on increasing eosinophil count.

Another important finding of this study is that a high concentration of Cu was associated with high WBC and eosinophil count. Further, synergistic effects of Cu and Mn on WBC, and Cu and As on eosinophil were observed. Several studies have explored the association between Cu poisoning and immune function 17, 18. Acute Cu intoxication, defined as oral intake > 10 mg Cu/L, can disrupt the gastrointestinal system 19. Ude et al. investigated the cytotoxicity of Cu^2+^ in an in vitro study^20^, in which they exposed undifferentiated Caco-2 intestinal cells to Cu(II) oxide (CuO) nanomaterials and Cu(II) sulfate (CuSO4) for 24 hours and then evaluated the effects. The results showed that both CuO nanomaterials and CuSO4 stimulated the secretion of IL-8 and impaired the viability of the undifferentiated Caco-2 cells 20. Their experiment suggests that Cu intoxication may play a role in immunomodulation. Another study by Ude et al. 21 explored the effects of CuO nanomaterials and CuSO4 on inflammatory mediators in an in vitro intestinal cell model. The results showed stimulation of reactive oxygen species (ROS), IL-8 secretion, and the heme oxygenase-1 gene, further suggesting an interaction between Cu and immune function 21. Our study is the first to provide clinical evidence showing a significant association between an elevated Cu level with both WBC and eosinophil count. In addition, this is the first clinical study to show significant synergistic effects between Cu and Mn on WBC, and Cu and As on eosinophil count. A possible explanation for these findings is that Cu intoxication leads to an increase in proinflammatory mediators and subsequent activation of immune responses, and that Cu may have synergistic effects with other heavy metals on immune function. Mn has been associated with neuroinflammation. 22, suggesting that it could cause an immune response. This could explain how the synergistic effect between Mn and Cu increased the WBC count. Moreover, since Cu and As had positive interaction effects on eosinophil count, our results show that two metals can have a synergistic effect on eosinophil count.

Another important finding of this study is that a high concentration of As was associated with a high eosinophil count. Exposure to As can lead to dysfunction of multiple organ systems, and even cancer 23. Blackfoot disease, an endemic vascular disease and a major health concern in southwest Taiwan in the early 20th century, was found to be caused by chronic As exposure through polluted water consumption 24. In addition, many animal studies have shown that As poisoning can impair the immune system. Nayak et al. 9 conducted a study in which zebrafish embryos were exposed to 2 to 10 ppb of sodium arsenate. After exposure, snakehead rhabdovirus was used to infect the zebrafish, and analysis of their immune reactions showed impaired innate immune function 9. Other studies have suggested that As poisoning may affect T cell differentiation 25, 26 and cell apoptosis 27, 28. However, little research has been conducted on the effect of As on eosinophil count. Chatterjee et al. 29 studied 120 children aged five to 15 years, of whom 68 were from As-contaminated areas and 52 were not. Analysis of cytogenic damage showed a significantly higher eosinophil count 29. Our findings are consistent with previous studies, in that As poisoning in Taiwan was also significantly associated with an elevated eosinophil count.

The final important finding of this study is that a high concentration of Cd was associated with a high eosinophil count. Cd exposure can occur through contaminated water, contaminated food, smoking, and occupational settings 30, 31. Cd exposure has been associated with bone degeneration 32, renal dysfunction 33, 34, impaired liver function 35, 36, cardiovascular disease 37, and malignancy 38. Several studies have explored the effects of Cd on cell apoptosis, autophagy, and ROS production 39, however few studies have explored its immunomodulation effects. Hu et al. reported Cd-induced proinflammatory activity in mouse placenta and human trophoblast cells after treatment with CdCl2, including elevated levels of TNF-α, IL-8 and IL-6 messenger RNA 40. In addition, Paniagua et al. conducted an in vitro study to explore the mechanisms of CdCl2 and the relationship with preeclampsia 41. In their experiment, JEG cells were treated with CdCl2 under different concentrations for 24 hours, and the results showed a dose-dependent increase in IL-6 through an ROS-associated mechanism. Our study is the first to show clinical evidence of an association between Cd and an elevated eosinophil count, indicating the potential role of Cd in allergic reactions that may be related to increased levels of proinflammatory mediators 41. Future studies are needed to investigate the association between Cd and immune function.

The main strength of this study is that we enrolled a large number of participants to explore the relationships among heavy metal and immune function. However, there were also several limitations. First, only single measurements of metal concentrations were made. Second, we used total As in urine as a measure of the exposure to the toxic form of inorganic As. Although total urine As can be measured quickly and is thus suitable when processing many samples, it cannot reflect differences in As metabolism and uptake between subjects. Nevertheless, total urine As is still used clinically and considered to be an acceptable biomarker to assess exposure to inorganic As. In addition, the participants were not asked about the environment near their homes, such as the presence of chemical plants, plastic factories, thermal power plants, gas stations, oil refineries and incinerators, which may have affected the heavy metal concentrations. Third, future studies should include data on differentiated WBC count to further investigate impaired innate and acquired immune systems. Finally, as this was a cross-sectional study, causal relationships and long-term clinical outcomes could not be ascertained. Long-term prospective studies with serial heavy metal measurements and immune function assessments are needed to verify our results.

In conclusion, in our analysis of a health survey of subjects residing in southern Taiwan, heavy metals such as Pb, As, Cu, and Cd were significantly associated with increased WBC and elevated eosinophil count. Further, synergistic effects of Pb and As and Mn and Cu on the association with WBC count, and As and Cu on the association with eosinophil count were observed. Our results show that heavy metals can have interaction effects on immune function, and offer clinical evidence of the immunomodulation effect of heavy metal exposure.

## Figures and Tables

**Table 1:** Comparison of clinical characteristics among participants according to WBC tertile

Characteristics	Tertile 1 (n = 815)	Tertile 2 (n = 820)	Tertile 3 (n = 822)	*p*
Age (year)	56.4 ± 12.8	55.8 ± 13.3	53.1 ± 13.2*†	< 0.001
Male gender (%)	30.6	41.1*	48.0*†	< 0.001
DM (%)	7.0	9.7	14.6*†	< 0.001
Hypertension (%)	23.3	26.0	26.7	0.252
BMI (kg/m^2^)	23.9 ± 3.4	25.1 ± 3.6*	26.1 ± 4.2*†	< 0.001
SBP (mmHg)	130.9 ± 20.4	131.9 ± 19.1	133.3 ± 19.7*	0.044
DBP (mmHg)	76.3 ± 12.1	77.7 ± 11.3	78.7 ± 11.6*	< 0.001
Laboratory parameters				
Fasting glucose (mg/dL)	96.1 ± 22.7	98.8 ± 23.5	104.8 ± 33.7*†	< 0.001
Triglyceride (mg/dL)	85 (62-125)	104 (74-148.75)*	122 (88-183.75)*†	< 0.001
Total cholesterol (mg/dL)	198.4 ± 36.5	200.4 ± 37.5	200.1 ± 38.4	0.508
HDL-cholesterol (mg/dL)	56.2 ± 13.7	53.4 ± 14.0*	49.4 ± 12.3*†	< 0.001
LDL-cholesterol (mg/dL)	116.1 ± 33.1	119.3 ± 33.6	122.1 ± 35.1*	0.002
WBC (*10^3^/μL)	4.5 ± 0.6	5.8 ± 0.4*	7.9 ± 1.4*†	< 0.001
Hemoglobin (g/dL)	13.5 ± 1.6	14.0 ± 1.6*	14.4 ± 1.6*†	< 0.001
Platelet (*10^3^/μL)	239.6 ± 63.3	258.4 ± 63.0*	282.5 ± 72.2*†	< 0.001
Eosinophil count (/μL)	60 (3.1-110)	80 (3.1-140)	80 (2.8-200)	0.078
eGFR (mL/min/1.73 m^2^)	88.8 ± 15.8	87.9 ± 16.8	90.5 ± 16.4†	0.004
Uric acid (mg/dL)	5.3 ± 1.4	5.8 ± 1.6*	6.0 ± 1.6*†	< 0.001
Heavy metals				
Blood				
Pb (mg/L)	1.5 (1.0-2.1)	1.5 (1.0-2.2)	1.6 (1.0-2.4)	0.079
Urine				
Ni (μg/L)	2.4 (1.5-3.6)	2.4 (1.6-3.8)	2.5 (1.6-3.7)	0.899
Cr (μg/L)	0.1 (0.1-0.1)	0.1 (0.1-0.1)	0.1 (0.1-0.1)	0.251
Mn (μg/L)	1.7 (1.0-3.0)	1.8 (0.9-2.9)	1.7 (0.9-2.9)	0.216
As (μg/L)	86.8 (48.7-159.9)	78.2 (45.3-134.6)*	73.7 (42.5-130.1)*	< 0.001
Cu (μg/dL)	1.3 (1.0-1.8)	1.5 (1.0-1.9)	1.6 (1.1-2.1)*†	< 0.001
Cd (μg/L)	0.9 (0.5-1.5)	0.8 (0.4-1.4)	0.8 (0.2-1.3)*	0.024

Abbreviations. WBC, white blood cell; DM, diabetes mellitus; BMI, body mass index; SBP, systolic blood pressure; DBP, diastolic blood pressure; HDL, high-density lipoprotein; LDL, low-density lipoprotein; eGFR, estimated glomerular filtration rate; Pb, lead; Ni, nickel; Cr, chromium; Mn, manganese; As, arsenic; Cu, copper; Cd, cadmium. The study patients were stratified into 3 groups according to tertiles of WBC. Tertile of WBC was defined as tertile 1: < 5.24*10^3^/μL, tertile 2: 5.24-6.49*10^3^/μL and tertile 3: ≥ 6.49*10^3^/μL. *p < 0.05 compared with tertile 2; †*p* < 0.05 compared with tertile 3.

**Table 2:** Association of heavy metals and WBC using multivariable linear regression analysis

Heavy metals	Multivariable (WBC)	
	Unstandardized coefficient β (95% CI)	*p*
Blood		
Pb (log per 1 mg/L)	0.332 (0.101, 0.562)	0.005
Urine		
Ni (log per 1 μg/L)	0.005 (-0.097, 0.106)	0.929
Cr (log per 1 μg/L)	0.215 (-0.147, 0.576)	0.244
Mn (log per 1 μg/L)	-0.111 (-0.226, 0.003)	0.057
As (log per 1 μg/L)	-0.006 (-0.183, 0.171)	0.948
Cu (log per 1 μg/dL)	0.476 (0.232, 0.721)	< 0.001
Cd (log per 1 μg/L)	-0.039 (-0.182, 0.103)	0.591

Values expressed as unstandardized coefficient β and 95% confidence interval (CI). Abbreviations are the same as in Table 1. Covariates in the multivariable model included age, sex, diabetes, hypertension, body mass index, systolic and diastolic blood pressures, fasting glucose, log triglyceride, total cholesterol, HDL-cholesterol, LDL-cholesterol, hemoglobin, platelet, eGFR and uric acid.

**Table 3:** Association of heavy metals and eosinophil count using multivariable linear regression analysis

Heavy metals	Multivariable (Eosinophil count)	
	Unstandardized coefficient β (95% CI)	*p*
Blood		
Pb (log per 1 mg/L)	0.732 (0.594, 0.869)	< 0.001
Urine		
Ni (log per 1 μg/L)	-0.006 (-0.068, 0.056)	0.840
Cr (log per 1 μg/L)	0.005 (-0.215, 0.226)	0.962
Mn (log per 1 μg/L)	0.013 (-0.056, 0.083)	0.706
As (log per 1 μg/L)	0.133 (0.025, 0.240)	0.015
Cu (log per 1 μg/dL)	0.181 (0.031, 0.330)	0.018
Cd (log per 1 μg/L)	0.139 (0.052, 0.226)	0.002

Values expressed as unstandardized coefficient β and 95% confidence interval (CI). Abbreviations are the same as in Table 1. Covariates in the multivariable model included age, sex, diabetes, hypertension, body mass index, systolic and diastolic blood pressures, fasting glucose, log triglyceride, total cholesterol, HDL-cholesterol, LDL-cholesterol, hemoglobin, platelet, eGFR and uric acid.
